# Distinct and shared risk factors for mood, anxiety and comorbid disorders among Canadians: evidence from the 2019–2020 Canadian Community Health Survey

**DOI:** 10.24095/hpcdp.46.5.03

**Published:** 2026-05

**Authors:** Fahima Hassan, Cindy Feng

**Affiliations:** 1 Department of Community Health and Epidemiology, Faculty of Medicine, Dalhousie University, Halifax, Nova Scotia, Canada

**Keywords:** mental health, anxiety disorder, mood disorder, comorbid conditions, comorbidity

## Abstract

**Introduction:**

Mood and anxiety disorders frequently co-occur, but few studies have differentiated their unique and shared risk factors. This study examines factors associated with mood disorders alone, anxiety disorders alone and comorbid mood and anxiety disorders among Canadians using 2019–2020 Canadian Community Health Survey data.

**Methods:**

The analytic sample included 107859 respondents, weighted to represent the Canadian population. Multinomial logistic regression with survey and bootstrap weights estimated adjusted relative risk ratios (aRRRs) for sociodemographic, socioeconomic, health-related and psychosocial factors.

**Results:**

Prevalence was 4.17% for mood disorders alone, 4.99% for anxiety disorders alone and 4.85% for comorbid mood and anxiety disorders. Females had significantly higher risks across all categories (comorbidity aRRR = 2.284; 95% confidence interval [CI]: 1.951–2.673). Younger adults (18–34 years) had greater risks for anxiety disorders alone (aRRR = 3.036; 95% CI: 2.441–3.776) and comorbid disorders (9.311; 7.134–12.153) compared with those aged 65 years and older. Lower household income and poor perceived health were consistently associated with increased risks, with comorbid disorders showing the strongest associations (poor perceived health aRRR = 14.688; 95% CI: 9.908–21.775). Psychosocial factors, including low life satisfaction and a weak sense of community belonging, were also linked to higher risks, particularly for comorbid disorders.

**Conclusion:**

Distinct and overlapping factors contribute to mood and/or anxiety disorders. Targeted prevention and intervention efforts addressing health status, socioeconomic disadvantage and psychosocial stressors—especially among younger people and females—are critical to reducing the burden of these mental health conditions in Canada.

**Highlights**

- Mood and anxiety disorder comorbidity is as prevalent as mood disorders alone or anxiety disorders alone, affecting nearly 5% of Canadians.
- Younger adults (18–34 years) and females have a significantly higher risk for all three mental health outcomes and especially for comorbid mood and anxiety disorders.
- Lower household income, poor perceived health and unmet health care needs are consistently and strongly associated with increased risk for mental health outcomes.
- Psychosocial factors like low life satisfaction and a weak sense of community belonging are linked to a higher risk for mood disorders or anxiety disorders, and particularly for co-occurring disorders.
- These findings highlight the need for targeted mental health interventions that address social, economic and psychosocial stressors.

## Introduction

Mood (e.g. depression, bipolar disorder) and anxiety disorders (e.g. generalized anxiety, panic disorder, phobias) are among the most common types of mental health conditions in Canada.^1^-^3^ Their effects on daily functioning and quality of life are substantial.^3^-^5^ The prevalence of diagnosed mood or anxiety disorders among people in Canada aged 12 years and older rose from 12% in 2015 to approximately 14% (equivalent to 4.4 million people) in 2019.^6^

A key complexity in mental health is the frequent co-occurrence of mood and anxiety disorders, known as psychiatric comorbidity.7 This phenomenon suggests shared underlying psychological and neurobiological mechanisms^7^-^10^ and heightened emotional reactivity.^11^ Additional evidence also points to distinct etiological pathways, symptom profiles and social determinants of these disorders.^6^,^8^-^10^ Mood disorders typically involve prolonged disturbances in mood, energy and motivation, whereas anxiety disorders are marked by excessive fear, worry and physiological arousal.^4^,^12^-^15^ Individuals with comorbid mood and anxiety disorders often experience more severe symptoms, longer illness duration and greater treatment resistance than those with either disorder alone.^16^ Aggregating mood and anxiety disorders into a single diagnostic category may therefore obscure important distinctions in risk factors and presentation.^17^ Understanding both the shared and unique correlates of mood and anxiety disorders is essential for improving diagnosis, tailoring treatment and guiding public health strategies.^18^

Mental health outcomes reflect a broad set of determinants: sociodemographic (e.g. sex, age, immigration status),^16^,^17^,^19^ socioeconomic (e.g. income and employment),^16^,^19^,^20^ health-related (e.g. multimorbidity, unmet health care needs)^17^,^19^ and psychosocial (e.g. stress, life satisfaction) factors.^17^,^19^ Many factors relate to both disorders,^11^ but some appear more specific: anxiety disorders have been associated with urban residence and childhood adversity, whereas mood disorders are more strongly linked to younger age, lower education level, being widowed or divorced and living in socioeconomically disadvantaged communities.^17^,^19^ Mood disorders are generally more prevalent among females.^3^,^21^ Multimorbidity, which is common among middle-aged and older adults, and unmet health care needs are especially relevant for mood disorders.^11^,^19^,^22^-^27^ Chronic pain frequently co-occurs with mood and anxiety disorders, as cause or consequence (e.g. arthritis, chronic back pain and chronic headaches). The differences reported across the provinces and territories in Canada likely reflect variations in health system organization, service access and social environments across regions.^3^ Finally, psychosocial factors like stress, poor perceived health and dissatisfaction with life are often modifiable, but cluster together and compound vulnerability.^28^,^29^

Despite growing research on mood and anxiety disorders in Canada, to the best of our knowledge, few studies have examined mood disorders alone, anxiety disorders alone and comorbid mood and anxiety disorders (henceforth referred to as “comorbid disorders”) in a single, nationally representative sample. Using the most recent pre-COVID-19 Canadian Community Health Survey (CCHS) data (2019–2020), we investigated a range of sociodemographic, socioeconomic, health-related and psychosocial factors associated with these three mental health outcomes. Our findings provide an up-to-date baseline for understanding mental health risk profiles and can inform targeted prevention and intervention strategies.

## Methods

***Data source and study population***

This study used pooled data from the 2019–2020 CCHS Annual Components, which were harmonized by Statistics Canada and analyzed as a single cross-sectional dataset.^30^ The CCHS uses a multistage, complex sampling design to collect comprehensive socioeconomic and health-related data, including information on health status, access to and utilization of health services, and various determinants of health.^30^-^32^ The survey sample included 108252 people aged 12 years and older living in all the provinces and territories in Canada. The survey does not include people living on reserves and other Indigenous (referred to as “Aboriginal” in the CCHS) settlements, in specific remote areas or institutional settings and full-time members of the Canadian Armed Forces. Detailed descriptions of the CCHS methodology, design, instruments and sampling frame are available elsewhere.^31^

***Study variables and measures***

**Outcome variable**

The primary outcome was a four-category multinomial variable representing no reported disorder, mood disorders alone, anxiety disorders alone and comorbid mood and anxiety disorders, based on self-reported chronic (lasting, or expected to last, at least 6 months) diagnoses of mood and/or anxiety disorders by a health professional. Respondents were categorized as having a mood disorder if they answered “yes” to the question “Do you have a mood disorder such as depression, bipolar disorder, mania or dysthymia?” and as having an anxiety disorder if they answered “yes” to the question “Do you have an anxiety disorder such as a phobia, obsessive-compulsive disorder or panic disorder?” Respondents who answered “yes” to the question about having a mood disorder and “no” to the question about having an anxiety disorder were categorized as having a mood disorder alone, whereas those who answered “yes” to the question about having an anxiety disorder and “no” to the question about having a mood disorder were categorized as having an anxiety disorder alone. Respondents who answered “yes” to both questions were categorized as having comorbid disorders, and those who answered “no” were categorized as having no reported disorder.

**Covariates**

The covariates included the following sociodemographic factors: sex (male or female); age group (12–17, 18–24, 25–34, 35–50, 51–64 or ≥65 years); Indigenous identity (yes or no); immigration status (immigrant [either a permanent resident, referred to as “landed immigrant” in the 2020 CCHS, or a nonpermanent resident] or Canadian born); racialized identity (referred to as “visible minority,” with yes or no indicators, in the CCHS public-use file, which collapses multiple self-identified ethnic and/or cultural categories into a binary indicator); marital status (married or living common law vs. single, i.e. never married, divorced, separated or widowed); educational attainment (less than high school graduation, high school diploma or equivalent, or postsecondary certificate, diploma or university degree); and region of residence (the 10 provinces and a combined territories category).

Socioeconomic factors included household income (< Canadian dollars [CAD] 20000, 20000–39999, 40000–59999, 60000–79999 or ≥ 80000), based on Canadian national income groupings; and household food security status (food secure, marginally insecure, moderately insecure or severely insecure).

Health-related factors included perceived health (excellent, very good, good, fair, poor); pain status (no usual pain or discomfort vs. usual pain or discomfort); number of chronic physical conditions (0, 1, 2 or ≥3 based on indicators for seven diagnosed conditions, i.e. asthma, arthritis, high blood pressure, diabetes, chronic respiratory diseases, musculoskeletal disorders and cardiovascular disease). Unmet health care need in the past 12 months (no/yes) was coded “yes” if the respondent reported needing but not receiving health care and experiencing one or more barriers to receiving health care (e.g. waiting time, cost, lack of availability).

Psychosocial factors included sense of community belonging (very strong, somewhat strong, somewhat weak or very weak); life satisfaction (very satisfied, satisfied, neither satisfied nor dissatisfied, dissatisfied or very dissatisfied); and perceived life stress (not at all stressful, not very stressful, a bit stressful, quite a bit stressful or extremely stressful).

Unless otherwise noted, covariates use Statistics Canada–derived variables in the 2019–2020 CCHS public-use file; nonresponse (“don’t know,” “refused”) was coded as missing. Some variables (e.g. chronic conditions count, unmet health care need) were constructed from multiple CCHS items.

**Conceptual framing**

Psychosocial measures (life satisfaction, perceived stress, sense of community belonging) are treated as downstream social determinants shaped by broader social and economic conditions (e.g. poverty, discrimination or exclusion). We therefore interpreted associations involving these variables as conditional associations that may reflect accumulated structural disadvantage rather than purely individual attributes.

***Statistical analysis***

Descriptive analyses summarize respondent characteristics across the levels of the outcome variable. We report frequencies and percentages using survey- and bootstrap-weighted estimates to account for the complex survey design. Crude associations were examined with bivariate multinomial logistic regression; adjusted associations were estimated with survey-weighted multinomial logistic regression to obtain adjusted relative risk ratios (aRRRs) and 95% confidence intervals (CIs). Associations were considered statistically significant if the 95% CI for each aRRR did not include 1. All analyses incorporated the CCHS-provided sampling weights to account for the complex survey design and to ensure population-level representativeness.^30^ Multicollinearity among explanatory variables was assessed using the variance inflation factor,^33^ with values exceeding 2.5 indicating potential multicollinearity concerns.

Analyses were performed using STATA version 17 (StataCorp LLC, College Station, TX, US).

***Ethics approval***

This study used publicly available, de-identified secondary data from the CCHS, and is therefore exempt from institutional ethics review.

## Results

In the analytic sample of 107859 respondents, 4503 (4.17%) reported being clinically diagnosed with a mood disorder alone, 5381 (4.99%) with an anxiety disorder alone and 5226 (4.85%) with comorbid mood and anxiety disorders (Table 1). Females were more prevalent in all the groups, and younger adults (18–34 years) were particularly represented in the anxiety alone and comorbid disorders groups.

**Table 1:** Characteristics of respondents with mood and/or anxiety disorders or no reported mood or anxiety disorder, CCHS, 2019–2020

Variables	No reported disorder		Mood disorders^b^		Anxiety disorders^c^		Comorbid mood and anxiety disorders^b^^c^		Total	
	n	%	n	%	n	%	n	%	n	%
**Total**	92 749	85.99	4503	4.17	5381	4.99	5226	4.85	107 859	100
Sociodemographic factors										
Sex (n = 107 859)										
Male	47 594	51.32	1834	40.73	1963	36.48	1878	35.93	53 269	49.39
Female	45 155	48.68	2669	59.27	3418	63.52	3348	64.07	54 590	50.61
Age, years (n = 107 859)										
12–17	6880	7.42	109	2.43	579	10.76	253	4.84	7822	7.25
18–34	23 415	25.25	1034	22.97	1713	31.83	2164	41.42	28 327	26.26
35–49	20 999	22.64	1044	23.19	1257	23.36	1253	23.97	24 553	22.76
50–64	21 920	23.63	1354	30.07	1106	20.56	1114	21.31	25 494	23.64
≥ 65	19 534	21.06	961	21.34	726	13.50	442	8.46	21 663	20.08
Indigenous identity (n = 104 880)										
No	87 814	97.36	4181	94.96	4996	95.49	4674	92.50	101 665	96.93
Yes	2378	2.64	222	5.04	236	4.51	379	7.50	3215	3.07
Immigration status (n = 105 728)										
Immigrant^d^	26 020	28.65	685	15.39	723	13.61	595	11.61	28 023	26.50
Canadian born	64 815	71.35	3769	84.61	4590	86.39	4532	88.39	77 705	73.50
Racialized identity^e^ (n = 104 902)										
Yes	21 659	24.04	487	11.02	597	11.30	575	11.29	23 317	22.23
No	68 453	75.96	3932	88.98	4684	88.70	4517	88.71	81 585	77.77
Marital status (n = 99 800)										
Married/living common law	55 775	65.11	2373	54.18	2616	54.56	2115	42.64	62 879	63.00
Single^f^	29 890	34.89	2007	45.82	2179	45.44	2845	57.36	36 921	37.00
Educational attainment (n = 102 176)										
Less than high school	3769	4.28	199	4.67	231	4.57	197	4.06	4396	4.30
High school graduate	10 634	12.08	608	14.26	582	11.52	817	16.85	12 642	12.37
Postsecondary graduate	73 605	83.63	3457	81.06	4239	83.90	3836	79.09	85 138	83.32
Region of residence (n = 107 859)										
Newfoundland and Labrador	1293	1.39	69	1.54	97	1.80	84	1.62	1543	1.43
Prince Edward Island	377	0.41	22	0.49	25	0.47	31	0.60	456	0.42
Nova Scotia	2236	2.41	159	3.52	171	3.18	253	4.84	2818	2.61
New Brunswick	1817	1.96	114	2.52	157	2.91	138	2.64	2225	2.06
Quebec	21 625	23.32	802	17.80	1437	26.71	637	12.20	24 501	22.72
Ontario	36 326	39.17	1747	38.80	2026	37.64	2273	43.50	42 372	39.28
Manitoba	3132	3.38	167	3.71	168	3.12	183	3.50	3649	3.38
Saskatchewan	2614	2.82	203	4.50	154	2.87	153	2.93	3124	2.90
Alberta	10 539	11.36	588	13.06	515	9.57	699	13.38	12 342	11.44
British Columbia	12 556	13.54	622	13.82	622	11.56	764	14.62	14 565	13.50
Territories^g^	234	0.25	11	0.23	10	0.18	10	0.19	264	0.24
Socioeconomic factors										
Household income, CAD (n = 106 620)										
< 20 000	3280	3.58	320	7.21	305	5.74	479	9.32	4384	4.11
20 000–39 999	9622	10.49	601	13.55	673	12.66	743	14.47	11 639	10.92
40 000–59 999	11 281	12.30	637	14.35	703	13.22	811	15.80	13 431	12.60
60 000–79 999	11 271	12.29	636	14.34	670	12.61	601	11.71	13 179	12.36
≥ 80 000	56 281	61.35	2243	50.55	2964	55.76	2500	48.69	63 987	60.01
Household food security status (n = 91 125)										
Food secure	71 675	91.54	3035	79.66	3914	83.85	3088	71.05	81 712	89.67
Marginally insecure	2591	3.31	213	5.60	226	4.84	230	5.29	3260	3.58
Moderately insecure	3062	3.91	325	8.52	337	7.23	538	12.37	4262	4.68
Severely insecure	973	1.24	237	6.22	191	4.09	491	11.29	1891	2.08
Health-related factors										
Perceived health (n = 107 725)										
Excellent	24 336	26.27	315	7.00	716	13.31	189	3.63	25 555	23.72
Very good	37 161	40.11	1190	26.47	1825	33.95	962	18.49	41 139	38.19
Good	23 871	25.76	1691	37.61	1931	35.91	1986	38.17	29 479	27.37
Fair	5655	6.10	948	21.09	683	12.70	1351	25.97	8638	8.02
Poor	1626	1.75	352	7.83	221	4.12	715	13.74	2914	2.71
Pain status (n = 107 570)										
No usual pain or discomfort	73 409	79.35	2513	56.16	3585	66.69	2802	53.87	82 310	76.52
Usual pain or discomfort	19 108	20.65	1962	43.84	1791	33.31	2400	46.13	25 260	23.48
Number of chronic physical conditions (n = 107 859)^h^										
0	56 801	61.24	1976	43.88	3058	56.82	2553	48.86	64 388	59.70
1	19 872	21.43	1194	26.51	1261	23.43	1429	27.34	23 755	22.02
2	9356	10.09	675	15.00	565	10.49	686	13.12	11 282	10.46
≥ 3	6719	7.24	658	14.62	498	9.25	559	10.69	8434	7.82
Unmet health care need in the past 12 months (n = 107 859)										
No	89 634	96.64	4145	92.06	5054	93.92	4451	85.17	103 285	95.76
Yes	3114	3.36	357	7.94	327	6.08	775	14.83	4574	4.24
Psychosocial factors										
Sense of community belonging (n = 103 408)										
Very strong	17 355	19.38	534	12.77	820	16.52	405	8.56	19 114	18.48
Somewhat strong	47 042	52.54	1902	45.48	2439	49.14	1913	40.43	53 296	51.54
Somewhat weak	20 005	22.34	1277	30.54	1234	24.86	1496	31.62	24 012	23.22
Very weak	5129	5.73	469	11.21	471	9.48	917	19.39	6986	6.76
Life satisfaction (n = 104 005)										
Very satisfied	39 076	43.38	788	18.75	1390	27.99	538	11.31	41 793	40.18
Satisfied	46 858	52.02	2620	62.31	3041	61.22	2762	58.06	55 281	53.15
Neither satisfied nor dissatisfied	2862	3.18	402	9.55	345	6.94	690	14.52	4299	4.13
Dissatisfied	1039	1.15	339	8.07	163	3.28	621	13.06	2162	2.08
Very dissatisfied	241	0.27	56	1.33	28	0.56	145	3.06	470	0.45
Perceived life stress (n = 107 478)										
Not at all stressful	14 010	15.16	273	6.08	256	4.77	158	3.06	14 697	13.67
Not very stressful	23 916	25.87	678	15.11	905	16.85	441	8.51	25 940	24.13
A bit stressful	38 262	41.39	1939	43.23	2367	44.05	1995	38.48	44 562	41.46
Quite a bit stressful	14 443	15.62	1287	28.69	1565	29.12	1962	37.83	19 256	17.92
Extremely stressful	1806	1.95	309	6.89	280	5.21	628	12.12	3023	2.81

**Abbreviations:** CAD, Canadian dollars; CCHS, Canadian Community Health Survey. 

^a^ Survey- and bootstrap-weighted distribution of estimates of frequencies and percentages. 

^b^ Mood disorders included depression, bipolar disorder, mania or dysthymia. 

^c^ Anxiety disorders included phobia, obsessive-compulsive disorder or panic disorder. 

^d^ Respondents were identified as immigrants if they self-identified as permanent residents (“landed immigrants” in the 2020 CCHS) or nonpermanent residents. 

^e^ Respondents were identified as racialized (“visible minority” in the 2020 CCHS public-use file) if they self-identified as South Asian, Chinese, Black, Filipino, Arab, Latin American, Southeast Asian, West Asian, Korean, Japanese or another category. 

^f^ Respondents were identified as single if they self-identified as divorced, separated, widowed or never married. 

^g^ Data from Yukon, Northwest Territories and Nunavut were combined due to small counts. 

^h^ Based on indicators for the following diagnosed conditions: asthma, arthritis, high blood pressure, diabetes, chronic respiratory diseases, musculoskeletal disorders and/or cardiovascular disease. 

The comorbid disorders groups generally exhibited greater social and health disadvantages, including higher proportions of respondents identifying as Indigenous, single or with poorer perceived health, usual pain or discomfort, unmet health care needs, weaker sense of community belonging, lower life satisfaction and higher perceived stress. Household food insecurity had substantial missing data (about 16700 respondents; 15.5%) and was excluded from the regression analyses to avoid reducing the analytic sample.

Table 2 shows unadjusted associations.

**Table 2:** Unadjusted univariate multinomial logistic regression associations between sociodemographic, socioeconomic, health-related and psychosocial factors and mental health outcomes among Canadians, CCHS, 2019–2020

Variables	Mood disorders^a^			Anxiety disorders^b^			Comorbid mood and anxiety disorders^a^^b^		
	RRR	95% CI	*p* value	RRR	95% CI	*p* value	RRR	95% CI	*p* value
Sociodemographic factors									
Sex (reference category: male)									
Female	1.534	1.378–1.708	< 0.001	1.835	1.641–2.053	< 0.001	1.879	1.670–2.114	< 0.001
Age, years (reference category: ≥ 65 years)									
12–17	0.324	0.232–0.451	< 0.001	2.264	1.883–2.722	< 0.001	1.626	1.267–2.085	< 0.001
18–34	0.898	0.766–1.053	0.187	1.967	1.705–2.270	< 0.001	4.085	3.539–4.715	< 0.001
35–49	1.011	0.878–1.165	0.879	1.610	1.405–1.845	< 0.001	2.637	2.271–3.061	< 0.001
50–64	1.256	1.108–1.423	< 0.001	1.357	1.179–1.562	< 0.001	2.246	1.928–2.616	< 0.001
Indigenous identity (reference category: no)									
Yes	1.961	1.586–2.425	< 0.001	1.743	1.408–2.157	< 0.001	2.994	2.428–3.692	< 0.001
Immigration status (reference category: immigrant^c^)									
Canadian born	2.208	1.885–2.586	< 0.001	2.548	2.140–3.034	< 0.001	3.057	2.565–3.644	< 0.001
Racialized identity^d^ (reference category: no)									
Yes	0.391	0.319–0.479	< 0.001	0.403	0.325–0.499	< 0.001	0.402	0.322–0.502	< 0.001
Marital status (reference category: married/living common law)									
Single^e^	1.578	1.418–1.756	< 0.001	1.554	1.385–1.744	< 0.001	2.510	2.251–2.799	< 0.001
Educational attainment (reference category: less than high school)									
High school graduate	1.082	0.874–1.339	0.469	0.893	0.720–1.107	0.302	1.471	1.127–1.919	0.004
Postsecondary graduate	0.888	0.743–1.062	0.194	0.939	0.794–1.111	0.465	0.998	0.779–1.277	0.986
Region of residence (reference category: Ontario)									
Newfoundland and Labrador	1.113	0.838–1.479	0.458	1.343	1.006–1.792	0.045	1.044	0.762–1.430	0.790
Prince Edward Island	1.212	0.861–1.707	0.270	1.203	0.852–1.697	0.294	1.329	0.983–1.798	0.065
Nova Scotia	1.474	1.161–1.872	0.001	1.371	1.105–1.700	0.004	1.807	1.457–2.241	< 0.001
New Brunswick	1.300	0.999–1.692	0.051	1.546	1.235–1.936	< 0.001	1.211	0.906–1.620	0.196
Quebec	0.771	0.651–0.913	0.003	1.192	1.041–1.365	0.011	0.471	0.397–0.559	< 0.001
Manitoba	1.110	0.872–1.412	0.397	0.960	0.758–1.217	0.739	0.932	0.741–1.172	0.547
Saskatchewan	1.612	1.310–1.981	< 0.001	1.058	0.785–1.427	0.709	0.936	0.715–1.226	0.631
Alberta	1.160	0.996–1.352	0.056	0.877	0.730–1.054	0.160	1.060	0.898–1.251	0.489
British Columbia	1.030	0.879–1.207	0.711	0.889	0.742–1.065	0.200	0.972	0.827–1.143	0.734
Territories^f^	0.937	0.725–1.210	0.616	0.731	0.562–0.953	0.020	0.675	0.515–0.886	0.005
Socioeconomic factors									
Household income, CAD (reference category: ≥ 80 000)									
< 20 000	2.449	2.034–2.949	< 0.001	1.766	1.440–2.166	< 0.001	3.285	2.795–3.862	< 0.001
20 000–39 999	1.568	1.353–1.817	< 0.001	1.328	1.133–1.557	< 0.001	1.738	1.487–2.031	< 0.001
40 000–59 999	1.416	1.217–1.649	< 0.001	1.183	0.999–1.400	0.052	1.619	1.370–1.914	< 0.001
60 000–79 999	1.416	1.203–1.667	< 0.001	1.130	0.959–1.331	0.145	1.201	1.011–1.427	0.037
Health-related factors									
Perceived health (reference category: excellent)									
Very good	2.478	1.997–3.074	< 0.001	1.670	1.415–1.971	< 0.001	3.340	2.522–4.424	< 0.001
Good	5.481	4.445–6.759	< 0.001	2.750	2.328–3.248	< 0.001	10.734	8.220–14.016	< 0.001
Fair	12.974	10.426–16.144	< 0.001	4.106	3.356–5.024	< 0.001	30.829	23.360–40.687	< 0.001
Poor	16.756	12.946–21.686	< 0.001	4.628	3.611–5.931	< 0.001	56.716	42.423–75.825	< 0.001
Pain status (reference category: no usual pain or discomfort)									
Usual pain or discomfort	2.999	2.698–3.333	< 0.001	1.919	1.722–2.138	< 0.001	3.290	2.954–3.665	< 0.001
Number of diagnosed chronic physical conditions^g^ (reference category: 0)									
1	1.727	1.506–1.980	< 0.001	1.179	1.035–1.343	0.014	1.599	1.399–1.828	< 0.001
2	2.075	1.790–2.406	< 0.001	1.121	0.968–1.298	0.128	1.630	1.391–1.911	< 0.001
≥ 3	2.817	2.429–3.265	< 0.001	1.376	1.173–1.615	< 0.001	1.850	1.571–2.177	< 0.001
Unmet health care needs (reference category: no)									
Yes	2.481	2.005–3.071	< 0.001	1.862	1.528–2.269	< 0.001	5.013	4.174–6.021	< 0.001
Psychosocial factors									
Sense of community belonging (reference category: very strong)									
Somewhat strong	1.314	1.120–1.542	0.001	1.097	0.942–1.277	0.233	1.742	1.451–2.092	< 0.001
Somewhat weak	2.075	1.745–2.468	< 0.001	1.305	1.101–1.547	0.002	3.205	2.651–3.875	< 0.001
Very weak	2.971	2.403–3.672	< 0.001	1.941	1.549–2.432	< 0.001	7.664	6.211–9.457	< 0.001
Life satisfaction (reference category: very satisfied)									
Satisfied	2.772	2.379–3.230	< 0.001	1.824	1.618–2.057	< 0.001	4.282	3.545–5.174	< 0.001
Neither satisfied nor dissatisfied	6.959	5.623–8.612	< 0.001	3.387	2.639–4.347	< 0.001	17.530	13.942–22.041	< 0.001
Dissatisfied	16.190	12.567–20.858	< 0.001	4.411	3.286–5.920	< 0.001	43.438	33.636–56.095	< 0.001
Very dissatisfied	11.449	7.304–17.947	< 0.001	3.250	2.035–5.190	< 0.001	43.785	29.385–65.240	< 0.001
Perceived life stress (reference category: not at all stressful)									
Not very stressful	1.456	1.156–1.833	0.001	2.069	1.673–2.559	< 0.001	1.632	1.140–2.338	0.007
A bit stressful	2.603	2.105–3.220	< 0.001	3.381	2.783–4.107	< 0.001	4.611	3.380–6.290	< 0.001
Quite a bit stressful	4.578	3.682–5.692	< 0.001	5.922	4.823–7.273	< 0.001	12.011	8.753–16.481	< 0.001
Extremely stressful	8.799	6.534–11.848	< 0.001	8.477	6.383–11.257	< 0.001	30.773	21.651–43.737	< 0.001

**Abbreviations:** RRR, relative risk ratio; CAD, Canadian dollars; CCHS, Canadian Community Health Survey; CI, confidence interval. 

^a^ Mood disorders included depression, bipolar disorder, mania or dysthymia. 

^b^ Anxiety disorders included phobia, obsessive-compulsive disorder or panic disorder. 

^c^ Respondents were identified as immigrants if they self-identified as permanent residents (“landed immigrants” in the 2020 CCHS) or nonpermanent residents. 

^d^ Respondents were identified as racialized (“visible minority” in the CCHS public-use file) if they self-identified as South Asian, Chinese, Black, Filipino, Arab, Latin American, Southeast Asian, West Asian, Korean, Japanese or another category. 

^e^ Respondents were identified as single if they self-identified as divorced, separated, widowed or never married. 

^f^ Values for Yukon, Northwest Territories and Nunavut were combined due to small counts. 

^g^ Based on indicators for the following diagnosed conditions: asthma, arthritis, high blood pressure, diabetes, chronic respiratory diseases, musculoskeletal disorders and/or cardiovascular disease. 

**Figure 1:**
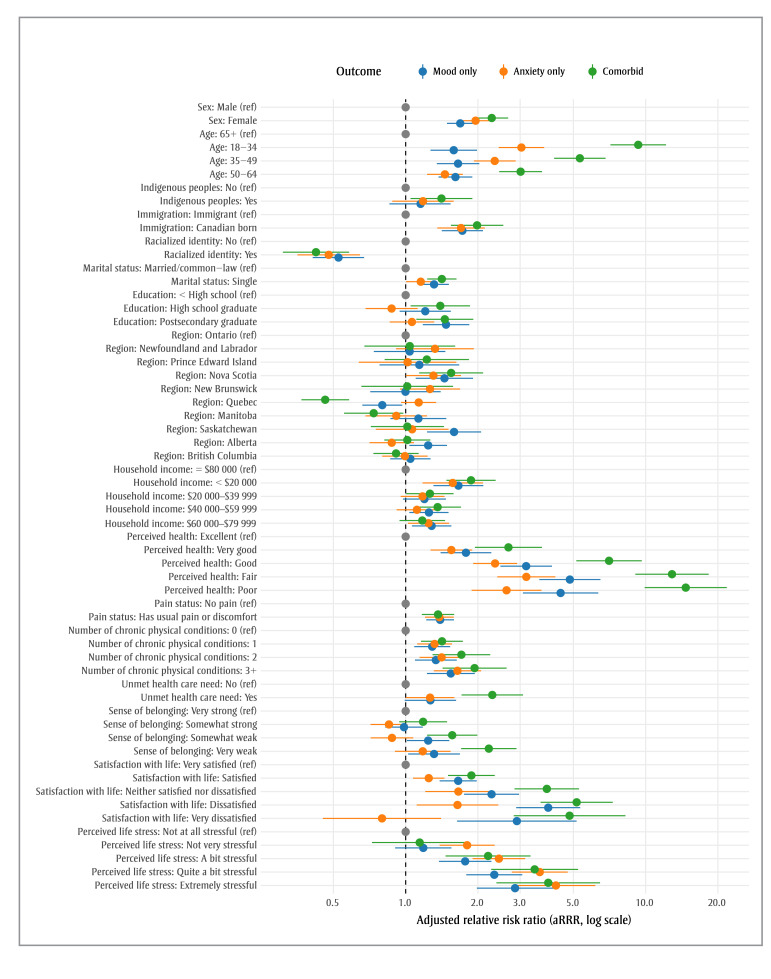
Adjusted relative risk ratios for mood, anxiety and comorbid disorders among Canadians, CCHS, 2019–2020

**Abbreviations: **CCHS, Canadian Community Health Survey; ref., reference. 

**Note: **Dots indicate value on the adjusted relative risk ratio axis. The bars on either side of each dot represent the associated 95% confidence interval. 

After adjustment, females had higher aRRRs for all three outcomes: mood disorders (aRRR = 1.688; 95% CI: 1.487–1.916); anxiety disorders (1.956; 1.712–2.234); and comorbid disorders (2.284; 1.951–2.673) in particular (Table 3 and Figure 1).

**Table 3:** Adjusted multinomial logistic regressiona associations between sociodemographic, socioeconomic, health-related and psychosocial factors and mental health outcomes among Canadians, CCHS, 2019–2020

Variables	Mood disorders^b^			Anxiety disorders^c^			Comorbid mood and anxiety disorders^b^^c^		
	aRRR	95% CI	*p* value	aRRR	95% CI	*p* value	aRRR	95% CI	*p* value
Sociodemographic factors									
Sex (reference category: male)									
Female	1.688	1.487–1.916	< 0.001	1.956	1.712–2.234	< 0.001	2.284	1.951–2.673	< 0.001
Age, years (reference category: ≥ 65 years)									
12–17	^d^	^d^	^d^	^d^	^d^	^d^	^d^	^d^	^d^
18–34	1.586	1.269–1.982	< 0.001	3.036	2.441–3.776	< 0.001	9.311	7.134–12.153	< 0.001
35–49	1.653	1.349–2.027	< 0.001	2.350	1.923–2.872	< 0.001	5.319	4.157–6.804	< 0.001
50–64	1.612	1.371–1.895	< 0.001	1.457	1.228–1.730	< 0.001	3.013	2.454–3.700	< 0.001
Indigenous identity (reference category: no)									
Yes	1.154	0.856–1.538	0.331	1.181	0.880–1.585	0.267	1.410	1.048–1.896	0.023
Immigration status (reference category: immigrant^e^)									
Canadian born	1.723	1.414–2.099	< 0.001	1.701	1.355–2.136	< 0.001	1.984	1.544–2.551	< 0.001
Racialized identity^f^ (reference category: no)									
Yes	0.525	0.410–0.671	< 0.001	0.478	0.354–0.645	< 0.001	0.423	0.308–0.581	< 0.001
Marital status (reference category: married/living common law)									
Single^g^	1.313	1.139–1.513	< 0.001	1.156	1.004–1.331	0.044	1.415	1.230–1.628	< 0.001
Educational attainment (reference category: less than high school)									
High school graduate	1.207	0.944–1.542	0.133	0.874	0.681–1.121	0.288	1.394	1.049–1.853	0.022
Postsecondary graduate	1.473	1.179–1.841	0.001	1.062	0.858–1.315	0.581	1.456	1.108–1.913	0.007
Region of residence (reference category: Ontario)									
Newfoundland and Labrador	1.039	0.737–1.463	0.829	1.325	0.913–1.923	0.139	1.040	0.673–1.608	0.859
Prince Edward Island	1.140	0.778–1.672	0.502	1.019	0.638–1.626	0.938	1.225	0.818–1.834	0.325
Nova Scotia	1.451	1.102–1.909	0.008	1.305	1.001–1.700	0.049	1.547	1.139–2.102	0.005
New Brunswick	0.998	0.712–1.399	0.989	1.263	0.947–1.686	0.112	1.015	0.654–1.575	0.948
Quebec	0.799	0.660–0.968	0.022	1.133	0.957–1.340	0.146	0.462	0.368–0.581	< 0.001
Manitoba	1.130	0.864–1.476	0.372	0.914	0.681–1.227	0.551	0.736	0.554–0.978	0.034
Saskatchewan	1.591	1.228–2.062	< 0.001	1.064	0.751–1.508	0.727	1.016	0.716–1.443	0.928
Alberta	1.241	1.036–1.486	0.019	0.875	0.707–1.082	0.217	1.016	0.815–1.267	0.885
British Columbia	1.046	0.863–1.269	0.646	0.993	0.799–1.234	0.950	0.912	0.734–1.133	0.404
Territories	^h^	^h^	^h^	^h^	^h^	^h^	^h^	^h^	^h^
Socioeconomic factors									
Household income, CAD (reference category: ≥ 80 000)									
< 20 000	1.659	1.306–2.106	< 0.001	1.571	1.176–2.098	0.002	1.872	1.479–2.369	< 0.001
20 000–39 999	1.197	0.974–1.471	0.087	1.176	0.953–1.452	0.131	1.262	1.005–1.583	0.045
40 000–59 999	1.250	1.036–1.509	0.020	1.114	0.917–1.352	0.276	1.359	1.086–1.700	0.007
60 000–79 999	1.283	1.062–1.550	0.010	1.245	1.022–1.517	0.029	1.173	0.943–1.459	0.152
Health-related factors									
Perceived health (reference category: excellent)									
Very good	1.783	1.398–2.274	< 0.001	1.550	1.270–1.892	< 0.001	2.681	1.944–3.696	< 0.001
Good	3.177	2.481–4.069	< 0.001	2.358	1.912–2.910	< 0.001	7.038	5.142–9.632	< 0.001
Fair	4.832	3.605–6.477	< 0.001	3.182	2.410–4.203	< 0.001	12.874	9.058–18.297	< 0.001
Poor	4.422	3.082–6.345	< 0.001	2.632	1.883–3.679	< 0.001	14.688	9.908–21.775	< 0.001
Pain status (reference category: no usual pain or discomfort)									
Usual pain or discomfort	1.392	1.219–1.590	< 0.001	1.382	1.204–1.586	< 0.001	1.364	1.168–1.592	< 0.001
Number of diagnosed chronic physical conditions^i^ (reference category: 0)									
1	1.290	1.086–1.532	0.004	1.320	1.117–1.560	0.001	1.417	1.159–1.731	0.001
2	1.336	1.094–1.632	0.005	1.412	1.143–1.745	0.001	1.707	1.295–2.251	< 0.001
≥ 3	1.542	1.227–1.939	< 0.001	1.643	1.310–2.062	< 0.001	1.937	1.425–2.633	< 0.001
Unmet health care need (reference category: no)									
Yes	1.268	0.991–1.622	0.059	1.264	1.001–1.595	0.049	2.296	1.710–3.083	< 0.001
Psychosocial factors									
Sense of community belonging (reference category: very strong)									
Somewhat strong	0.984	0.823–1.178	0.861	0.852	0.714–1.017	0.076	1.182	0.940–1.487	0.153
Somewhat weak	1.241	1.013–1.520	0.037	0.877	0.715–1.075	0.206	1.563	1.229–1.988	< 0.001
Very weak	1.313	1.024–1.683	0.032	1.178	0.902–1.539	0.229	2.221	1.703–2.895	< 0.001
Life satisfaction (reference category: very satisfied)									
Satisfied	1.655	1.386–1.977	< 0.001	1.248	1.074–1.450	0.004	1.879	1.502–2.350	< 0.001
Neither satisfied nor dissatisfied	2.280	1.752–2.968	< 0.001	1.658	1.207–2.279	0.002	3.874	2.844–5.275	< 0.001
Dissatisfied	3.926	2.889–5.337	< 0.001	1.644	1.112–2.430	0.013	5.157	3.648–7.289	< 0.001
Very dissatisfied	2.906	1.638–5.156	< 0.001	0.797	0.452–1.404	0.432	4.819	2.821–8.232	< 0.001
Perceived life stress (reference category: not at all stressful)									
Not very stressful	1.184	0.904–1.551	0.221	1.803	1.385–2.348	< 0.001	1.144	0.724–1.807	0.564
A bit stressful	1.770	1.376–2.276	< 0.001	2.446	1.902–3.145	< 0.001	2.205	1.467–3.313	< 0.001
Quite a bit stressful	2.340	1.789–3.061	< 0.001	3.620	2.769–4.733	< 0.001	3.447	2.272–5.229	< 0.001
Extremely stressful	2.856	1.981–4.116	< 0.001	4.228	2.896–6.172	< 0.001	3.927	2.390–6.453	< 0.001

**Abbreviations:** aRRR, adjusted relative risk ratio; CAD, Canadian dollars; CCHS, Canadian Community Health Survey; CI, confidence Interval. 

^a^ aRRRs and 95% CIs from survey- and bootstrap-weighted multinomial logistic regression analyses. 

^b^ Mood disorders included depression, bipolar disorder, mania or dysthymia. 

^c^ Anxiety disorders included phobia, obsessive-compulsive disorder or panic disorder. 

^d^ aRRRs for the 12–17-year age group are not reported because collinearity with marital status was perfect as all respondents were single, which prevented unique parameter estimation. 

^e^ Respondents who self-identified as permanent residents (referred to as “landed immigrants” in the 2020 CCHS) or nonpermanent residents. 

^f^ Respondents were identified as racialized (“visible minority” in the CCHS public-use file) if they self-identified as South Asian, Chinese, Black, Filipino, Arab, Latin American, Southeast Asian, West Asian, Korean, Japanese or another category. 

^g^ Respondents were identified as single if they self-identified as divorced, separated, widowed or never married. 

^h^ aRRRs for Yukon, Northwest Territories and Nunavut combined are suppressed due to very small cell counts (mood disorders, n = 11; anxiety disorders, n = 10; comorbid disorders, n = 10) and likely instability; descriptive estimates are shown in Table 1. 

^i^ Based on indicators for the following diagnosed chronic conditions: asthma, arthritis, high blood pressure, diabetes, chronic respiratory diseases, musculoskeletal disorders and/or cardiovascular disease. 

Age was strongly associated with mental health outcomes: young adults (18–34 years) had more than three times the risk for anxiety disorders (aRRR = 3.036; 95% CI: 2.441–3.776) and more than nine times the risk for comorbid disorders (9.311; 7.134–12.153) than older adults (≥65 years). Adults aged 35 to 64 years also had higher risks across all outcomes, though to a lesser extent than younger adults.

We tested an exploratory age-by-sex interaction term in the multinomial model; the global Wald test was not statistically significant (χ^2^(12) = 7.82; *p* = 0.80), indicating that the associations between sex and mental health outcomes did not differ significantly across age groups, contrary to previous Canadian surveillance findings that indicated higher mental health burden among younger females.^3^

Indigenous identity was associated with higher comorbid disorders (aRRR = 1.410; 95% CI: 1.048–1.896), but not with mood and anxiety disorders individually. Canadian-born respondents had higher risks than immigrants, particularly for comorbid disorders (1.984; 1.544–2.551). Racialized respondents were significantly less likely than nonracialized respondents to report mood disorders alone (0.525; 0.410–0.671), anxiety disorders (0.478; 0.354–0.645) and comorbid disorders (0.423; 0.308–0.581). Single respondents had higher risks across all outcomes compared with those who were married or in common-law relationships.

Compared with respondents with less than high school education, postsecondary graduates had higher risks for mood disorders alone (aRRR = 1.473; 95% CI: 1.179–1.841) and comorbid disorders (1.456; 1.108–1.913). High school graduates had a significantly increased risk for comorbid disorders (1.394; 1.049–1.853).

Regional differences in risks for mood and/or anxiety disorders were significant across five provinces. Relative to those in Ontario, respondents in Saskatchewan (aRRR = 1.591; 95% CI: 1.228–2.062) and Alberta (1.241; 1.036–1.486) showed a higher risk for mood disorders alone. Those in Nova Scotia had greater risks for comorbid disorders (1.547; 1.139–2.102) and anxiety disorders alone (1.305; 1.001–1.700). Conversely, respondents in Quebec had lower risks for mood disorders alone (0.799; 0.660–0.968) and comorbid disorders (0.462; 0.368–0.581) and those in Manitoba had lower risk for comorbid disorders (0.736; 0.554–0.978). Estimates for the territories combined are not reported due to very small cell sizes.

Lower income was linked to higher risks, especially for those with an income less thanCAD 20000 (highest risk across the disorder categories). Mid-income effects were mixed and often nonsignificant for these diagnosed with anxiety disorders alone.

Poorer perceived health showed a strong gradient that was largest for comorbid disorders (aRRR of 2.681 [95% CI: 1.944–3.696] for very good perceived health to 14.688 [9.908–21.775] for poor perceived health). Experiencing usual pain or discomfort was associated with higher risk for mood (1.392; 1.219–1.590), anxiety (1.382; 1.204–1.586) and comorbid disorders (1.364; 1.168–1.592). Risk rose with the number of coexisting chronic conditions, with three or more associated with higher risks for mood (1.542; 1.227–1.939), anxiety (1.643; 1.310–2.062) and comorbid disorders (1.937; 1.425–2.633). Having unmet health care needs was associated with comorbid disorders (2.296; 1.710–3.083) and anxiety disorders alone (1.264; 1.001–1.595), but not with mood disorders alone.

Psychosocial factors were strongly associated with the outcomes. Compared with having a very strong sense of community belonging, a very weak sense of community belonging was linked to higher risks for mood disorders alone (aRRR = 1.313; 95% CI: 1.024–1.683) and comorbid disorders (2.221; 1.703–2.895). Life satisfaction was strongly associated with mental health outcomes. Compared with respondents who were very satisfied with life, progressively lower levels of life satisfaction were associated with higher risks of mood disorders and comorbid disorders with a clear dose–response pattern. Associations with anxiety disorders were present but generally weaker and less consistent.

Perceiving life stress demonstrated a clear dose–response relationship with mental health outcomes. Increasing levels of stress were associated with progressively higher risks of anxiety disorders and comorbid disorders, with the strongest associations observed among respondents reporting extreme stress. Associations with mood disorders were weaker at lower stress levels but became more pronounced at moderate to high levels of perceived stress. 

## Discussion

This study provides a prepandemic profile of factors associated with mood disorders, anxiety disorders and comorbid mood and anxiety disorders among adolescents (≥ 12 years) and adults in Canada, using nationally representative data from the 2019 to 2020 CCHS. We found that 4.17% of respondents reported receiving a clinical diagnosis of a mood disorder, 4.99% of an anxiety disorder and 4.85% of comorbid disorders. While the prevalence of mood and anxiety disorders individually is broadly consistent with earlier research,^16^ the similar prevalence of comorbid disorders underscores the clinical and public health importance of co-occurring mental health conditions.

Our results confirm well-established sociodemographic patterns. Females had significantly higher relative risks, compared with males, across all mental health outcomes, with the strongest association for comorbid disorders (aRRR of 2.28 vs. 1.69 for mood disorders alone and 1.96 for anxiety disorders alone). This aligns with Canadian and global evidence showing greater prevalence of mood and anxiety disorders among females.^3^,^21^ Likely contributors include biological differences in stress regulation, greater exposure to interpersonal stressors and gendered norms around emotional expression and help-seeking. Disproportionate caregiving roles and gendered socioeconomic disadvantage may further heighten chronic stress and risk for comorbidity risk.^22^ In contrast, lower reported prevalence among males may partly reflect underdiagnosis and reluctance to disclose distress due to norms around stoicism and self-reliance.^16^ Together, these patterns suggest that gendered social and structural determinants intersect with biology to shape disparities in mental health.

Compared with older adults (≥65 years), younger adults and particularly those aged 18 to 34 years had markedly higher risks for anxiety disorders alone and comorbid disorders, with a striking nearly nine-fold increase in risk for comorbidity. These findings align with prior Canadian and international studies, and likely reflect age-related differences in stress exposure, socioeconomic pressures, coping resources and help-seeking.^18^,^19^ The findings also highlight the need for age-specific, low-barrier mental health supports for youth and young adults (e.g. campus-based services, youth-appropriate virtual care, brief counselling).

Individuals who identified as Indigenous had higher risk for comorbid mental health disorders (aRRR = 1.41). This is consistent with previous research findings^34^ and likely reflects the enduring impacts of colonialism, intergenerational trauma, systemic inequities and underlying social determinants of health such as poverty, housing insecurity and limited access to culturally safe care.^35^ However, the CCHS excludes people living on reserves, in Indigenous settlements and in many remote communities as well as in institutions,^31^ which likely means our results underestimate the true burden of mental health disorders among Indigenous people. Because population surveys cannot fully capture the historical and structural determinants of Indigenous mental health, national surveillance and service planning should be conducted, in partnership with Indigenous organizations, in ways that respect Indigenous data sovereignty and support culturally grounded, community-led care. 

Canadian-born respondents had higher risks across all mental health outcomes than immigrants. While this pattern is often described as consistent with the “healthy immigrant effect,”^36^-^38^ its applicability to mental health outcomes warrants careful interpretation. The lower prevalence observed among immigrants may reflect underutilization of mental health services, stigma related to help-seeking and barriers to accessing care rather than true differences in underlying mental health burden.^39^-^41^ Moreover, mental health risks vary substantially across immigrant groups, with refugees and individuals with forced migration experiences potentially experiencing greater risk than suggested by aggregated immigrant categories.^42^,^43^

Racialized respondents had lower relative risks for mood disorders, anxiety disorders and comorbid conditions (aRRRs = 0.423–0.525). While this may reflect protective cultural or familial support networks that buffer psychological distress, it too should be interpreted with caution. Lower reported prevalence among racialized populations may also arise from underdiagnosis and underreporting linked to stigma, limited access to culturally safe services and barriers to mental health assessment.^44^,^45^ The CCHS public-use file collapses different racialized identities into a binary “visible minority” indicator, which obscures heterogeneity and likely masks disparities between specific racialized groups.^46^ These findings reinforce the need for disaggregated analyses by race, ethnicity and immigration status and for culturally responsive measurement.

Education showed a more complex relationship. A completed postsecondary education was associated with increased risks for mood disorders and comorbid conditions, but not with anxiety disorders. This is not entirely consistent with earlier work that suggested a straightforward protective effect of education on mental health,^11^ but it does align with Finnish and Canadian studies that have found no consistent protection of higher education against anxiety disorders.^19^,^47^ One plausible explanation is that individuals with higher educational attainment may have greater mental health literacy, better access to primary care and more opportunities to receive and report a formal diagnosis of depression or a mood disorder. Conversely, work–family strain, job insecurity within professionalized labour markets and chronic work stress may contribute to mood symptoms despite higher educational attainment.

We observed differences between the provinces: compared with Ontario, Saskatchewan had the highest adjusted risk for mood disorders and Nova Scotia for comorbid conditions, while Quebec showed lower risks across most outcomes, consistent with previous research.^3^,^17^ These patterns likely reflect unmeasured contextual factors—primary-care attachment and service organization (e.g. availability of stepped care, waiting times, rural access), social policy environments (income, housing, employment supports) and help-seeking or diagnostic practices (literacy, stigma, screening, billing/coding). Such factors may be especially relevant for comorbidity, which often requires more coordinated care pathways.

Lower household income was positively associated with all mental health outcomes even after adjustment, consistent with evidence linking socioeconomic disadvantage to depression and anxiety.^48^-^51^ Our results underscore the continued importance of socioeconomic factors in mental health disparities and suggest that income remains a relevant consideration for targeted prevention and intervention strategies.

Finally, several health-related and psychosocial factors—poor perceived health and experiencing chronic pain, multimorbidity, unmet health care needs, dissatisfaction with life and higher stress—were strongly associated with all outcomes. Poor perceived health had the strongest association with comorbid disorders, suggesting heavier overall symptom burden and lower perceived capacity for self-management. Unmet health care needs may reflect structural and stigma-related barriers to timely mental health care.^24^,^52^,^53^

Having a weaker sense of community belonging was associated with higher risks for mood disorder and comorbidity, consistent with prior work linking low sense of community belonging to poorer mental health and higher risk for depression.^54^,^55^ Rather than an individual trait, having a weaker sense of community belonging can indicate structural disconnection driven by poverty, exclusion and discrimination that undermines social cohesion and resilience. Large-scale CCHS analyses have also shown an inverse relationship between life satisfaction and mental illness, independent of income, health or gender.^28^,^29^

Taken together, these patterns are consistent with the social determinants of health and the socioecological frameworks^56^,^57^ in which community belonging, stress and life satisfaction reflect upstream social and environmental contexts that shape exposure to—and coping with—psychological distress. We therefore interpret these psychosocial measures as markers and potential mediators of accumulated disadvantage, not merely individual attributes. While avoiding causal claims in this cross-sectional design, this framing helps explain why associations are strongest for comorbidity and suggests that linking social supports (e.g. income assistance, housing, community-connection programs such as social-prescribing initiatives) with clinical care may be especially relevant for people with multiple co-occurring needs.

***Strengths and limitations***

This study uses the large, nationally representative CCHS to examine population-level associations between sociodemographic, socioeconomic, psychosocial and health-related factors and mood and anxiety disorders among people living in Canada. Although the data were collected before the COVID-19 pandemic and may not reflect current mental health trends, it provides a useful snapshot of prepandemic mental health that can serve as a point of comparison for future studies. By distinguishing mood and anxiety disorders and comorbid outcomes and applying survey and bootstrap weights, this analysis offers a nuanced understanding of shared and distinct correlates.

Several limitations should be noted. The CCHS likely underestimates the true burden of mood and anxiety disorders because it relies on self-reported professionally diagnosed conditions and excludes undiagnosed or undisclosed cases. Its cross-sectional design precludes causal inference. The survey also excludes people living on reserves, in remote regions and in institutional settings, potentially resulting in underrepresentation of population groups facing structural inequities. In addition, the public-use file collapses diverse racialized identities into a binary “visible minority” variable, masking heterogeneity across groups. Important factors such as family history were unavailable.

Finally, several covariates (e.g. perceived health, multimorbidity, stress, life satisfaction) may act as mediators rather than independent predictors; adjusted estimates should therefore be interpreted as conditional associations. Future longitudinal and linkage studies could validate self-reports, assess temporality and better capture the mental health needs of excluded and marginalized populations.

## Conclusion

This study identifies shared and distinct associations with mood disorders, anxiety disorders and their comorbidity in Canada. Higher relative risk was observed among younger adults, females and those with lower income, poorer perceived health, multimorbidity, unmet health care needs and adverse psychosocial profiles (dissatisfaction with life, weak sense of community belonging, higher stress). Individuals with comorbid mood and anxiety conditions exhibited the greatest overall burden across clinical and social indicators. While not causal, these patterns can inform service planning: age-tailored, low-barrier supports for youth and young adults; culturally grounded, community-led approaches for Indigenous people; and care models that link social supports (e.g. income, housing, community-connection programs) with clinical services for people reporting unmet needs or social isolation. Coordinated, team-based and culturally safe care (e.g. collaborative or stepped care) in primary and community settings may be particularly relevant for those with co-occurring conditions. Ensuring timely, equitable and culturally safe access to care remains a central priority.

## Acknowledgements

We sincerely thank the Editor, Associate Editor and the two anonymous reviewers for their constructive and insightful comments, which substantially improved the clarity and rigour of this manuscript. We are additionally grateful to the English Editor, Joanna Odrowaz, as well as the French Editor, Anna Olivier, for their thorough, thoughtful and highly valuable editorial reviews.

## Funding

This work was supported by Research Nova Scotia through the New Health Investigator Grant.

## Conflicts of interest

The authors declare no conflicts of interest.

## Authors’ contributions and statement

FH: Conceptualization, methodology, data curation, formal analysis, writing—original draft; writing—review and editing. CF: Conceptualization, methodology, data curation, formal analysis, visualization, funding acquisition, project administration, supervision, validation, writing—review and editing.

The authors have read and approved the final manuscript and agree to be accountable for all aspects of the work.

The content and views expressed in this article are those of the authors and do not necessarily reflect those of the Government of Canada.
